# Supplementary material for: Pyruvate Dehydrogenase Kinase 1 inhibition mediated oxidative phosphorylation enhancement in cartilage promotes osteoarthritis progression
Source: BMC Musculoskelet Disord. 2023 Jul 20;24:597. doi: 10.1186/s12891-023-06585-6 (PMC10357736; doi:10.1186/s12891-023-06585-6)
Supplement: Supplementary file 1 — Additional file 1. [file 12891_2023_6585_MOESM1_ESM.zip › MF.Gsea.1653627316242/GOMF_NEUROPEPTIDE_RECEPTOR_BINDING.html]

Details for gene set GOMF\_NEUROPEPTIDE\_RECEPTOR\_BINDING[GSEA]

|  || Dataset | 55457\_collapsed\_to\_symbols.cls.cls #OA\_versus\_normal.cls.cls #OA\_versus\_normal\_repos |
| Phenotype | cls.cls#OA\_versus\_normal\_repos |
| Upregulated in class | normal |
| GeneSet | GOMF\_NEUROPEPTIDE\_RECEPTOR\_BINDING |
| Enrichment Score (ES) | -0.43351233 |
| Normalized Enrichment Score (NES) | -1.4026815 |
| Nominal p-value | 0.08235294 |
| FDR q-value | 0.26501858 |
| FWER p-Value | 1.0 |
Table: GSEA Results Summary

  

Fig 1: Enrichment plot: GOMF\_NEUROPEPTIDE\_RECEPTOR\_BINDING      
 Profile of the Running ES Score & Positions of GeneSet Members on the Rank Ordered List

  

| SYMBOL | TITLE | RANK IN GENE LIST | RANK METRIC SCORE | RUNNING ES | CORE ENRICHMENT || 1 | TAC1 | tachykinin precursor 1 [Source:HGNC Symbol;Acc:HGNC:11517] | 1219 | 0.473 | -0.0306 | No |
| 2 | ASIP | agouti signaling protein [Source:HGNC Symbol;Acc:HGNC:745] | 2596 | 0.291 | -0.0980 | No |
| 3 | ADCYAP1 | adenylate cyclase activating polypeptide 1 [Source:HGNC Symbol;Acc:HGNC:241] | 4398 | 0.121 | -0.2215 | No |
| 4 | SHANK1 | SH3 and multiple ankyrin repeat domains 1 [Source:HGNC Symbol;Acc:HGNC:15474] | 4511 | 0.112 | -0.2150 | No |
| 5 | NMU | neuromedin U [Source:HGNC Symbol;Acc:HGNC:7859] | 4578 | 0.107 | -0.2056 | No |
| 6 | NMB | neuromedin B [Source:HGNC Symbol;Acc:HGNC:7842] | 4611 | 0.104 | -0.1940 | No |
| 7 | NTS | neurotensin [Source:HGNC Symbol;Acc:HGNC:8038] | 4649 | 0.100 | -0.1833 | No |
| 8 | PYY | peptide YY [Source:HGNC Symbol;Acc:HGNC:9748] | 5499 | 0.033 | -0.2449 | No |
| 9 | GAL | galanin and GMAP prepropeptide [Source:HGNC Symbol;Acc:HGNC:4114] | 5599 | 0.024 | -0.2493 | No |
| 10 | GNAO1 | G protein subunit alpha o1 [Source:HGNC Symbol;Acc:HGNC:4389] | 6631 | -0.052 | -0.3223 | No |
| 11 | PPY | pancreatic polypeptide [Source:HGNC Symbol;Acc:HGNC:9327] | 7157 | -0.092 | -0.3506 | No |
| 12 | NPY | neuropeptide Y [Source:HGNC Symbol;Acc:HGNC:7955] | 7834 | -0.143 | -0.3836 | No |
| 13 | PMCH | pro-melanin concentrating hormone [Source:HGNC Symbol;Acc:HGNC:9109] | 8038 | -0.158 | -0.3780 | No |
| 14 | EDN3 | endothelin 3 [Source:HGNC Symbol;Acc:HGNC:3178] | 8754 | -0.213 | -0.4047 | Yes |
| 15 | GNAS | GNAS complex locus [Source:HGNC Symbol;Acc:HGNC:4392] | 8929 | -0.226 | -0.3875 | Yes |
| 16 | PRLH | prolactin releasing hormone [Source:HGNC Symbol;Acc:HGNC:17945] | 9443 | -0.268 | -0.3910 | Yes |
| 17 | GHRH | growth hormone releasing hormone [Source:HGNC Symbol;Acc:HGNC:4265] | 9533 | -0.277 | -0.3604 | Yes |
| 18 | HCRT | hypocretin neuropeptide precursor [Source:HGNC Symbol;Acc:HGNC:4847] | 10309 | -0.352 | -0.3729 | Yes |
| 19 | CCKBR | cholecystokinin B receptor [Source:HGNC Symbol;Acc:HGNC:1571] | 10842 | -0.410 | -0.3587 | Yes |
| 20 | EDN2 | endothelin 2 [Source:HGNC Symbol;Acc:HGNC:3177] | 10968 | -0.427 | -0.3106 | Yes |
| 21 | AGRP | agouti related neuropeptide [Source:HGNC Symbol;Acc:HGNC:330] | 10994 | -0.429 | -0.2544 | Yes |
| 22 | POMC | proopiomelanocortin [Source:HGNC Symbol;Acc:HGNC:9201] | 11010 | -0.431 | -0.1971 | Yes |
| 23 | EDN1 | endothelin 1 [Source:HGNC Symbol;Acc:HGNC:3176] | 11276 | -0.468 | -0.1543 | Yes |
| 24 | NPPA | natriuretic peptide A [Source:HGNC Symbol;Acc:HGNC:7939] | 11653 | -0.525 | -0.1123 | Yes |
| 25 | UCN | urocortin [Source:HGNC Symbol;Acc:HGNC:12516] | 12536 | -0.765 | -0.0771 | Yes |
| 26 | KISS1 | KiSS-1 metastasis suppressor [Source:HGNC Symbol;Acc:HGNC:6341] | 12556 | -0.776 | 0.0267 | Yes |
Table: GSEA details [plain text format]

  

Fig 2: GOMF\_NEUROPEPTIDE\_RECEPTOR\_BINDING      
 Blue-Pink O' Gram in the Space of the Analyzed GeneSet

  

Fig 3: GOMF\_NEUROPEPTIDE\_RECEPTOR\_BINDING: Random ES distribution      
 Gene set null distribution of ES for **GOMF\_NEUROPEPTIDE\_RECEPTOR\_BINDING**
